# Supplementary figures and images for: Iron overload inhibits BMP/SMAD and IL-6/STAT3 signaling to hepcidin in cultured hepatocytes
Source: PLoS One. 2021 Jun 23;16(6):e0253475. doi: 10.1371/journal.pone.0253475 (PMC8221488; doi:10.1371/journal.pone.0253475)

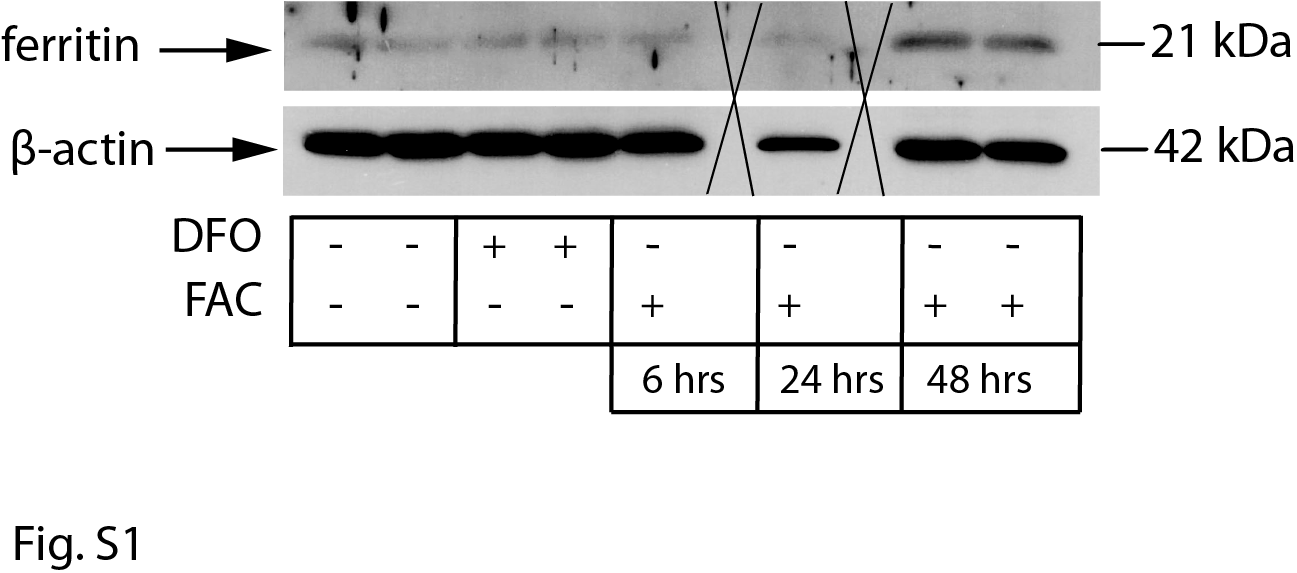

Supplement: S1 Fig — Huh7 cells were treated with either 100 μM DFO for 18 hours or 50 μM FAC for the indicated time intervals. Ferritin and β-actin expression in cell lysates were assessed by Western blotting. Western data are representative of two independent experiments. (TIF) [file pone.0253475.s001.tif]
