# Supplementary material for: Iron overload inhibits BMP/SMAD and IL-6/STAT3 signaling to hepcidin in cultured hepatocytes
Source: PLoS One. 2021 Jun 23;16(6):e0253475. doi: 10.1371/journal.pone.0253475 (PMC8221488; doi:10.1371/journal.pone.0253475)
Supplement: S1 Table — (DOCX) [file pone.0253475.s002.docx]

| S1 Table. List of primers used for qPCR. | | |  | |
| --- | --- | --- | --- | --- |
| Gene | GenBank accession | Forward primer sequence | | Reverse primer sequence |
|  |  |  | |  |
| Hamp1 | NM_032541.1 | *AAGCAGGGCAGACATTGCGAT* | | *CAGGATGTGGCTCTAGGCTATGT* |
| Tfrc | NM_011638.4 | *AGCCAGATCAGCATTCTCTAACT* | | *GCCTTCATGTTATTGTCGGCAT* |
| Bmp6 | NM_007556.2 | *ACTCGGGATGGACTCCACGTCA* | | *CACCATGAAGGGCTGCTTGTCG* |
| Id1 | NM_010495.2 | *GGTACTTGGTCTGTCGGAGC* | | *GCAGGTCCCTGATGTAGTCG* |
| Smad7 | NM_001042660.1 | *CGGACAGCTCAATTCGGAC* | | *GGTAACTGCTGCGGTTGTAA* |
| Socs3 | NM_007707.3 | *TGCGCCTCAAGACCTTCAG* | | *GCTCCAGTAGAATCCGCTCTC* |
| Rpl19 | NM_009078.2 | *AGGCATATGGGCATAGGGAAGAG* | | *TTGACCTTCAGGTACAGGCTGTG* |
| HAMP | NM_021175 | *ATGGCACTGAGCTCCCAGAT* | | *ACTTTGATCGATGACAGCAG* |
| RPS18 | NM_022551 | *TGTGGTGTTGAGGAAAGCAG* | | *AAGTGACGCAGCCCTCTATG* |
| GCLC | NM_001498.4 | *ACAGCCCTACGGAGGAACAA* | | *GTGAACCCAGGACAGCCTAA* |
| ID1 | NM_002165.4 | *TTACTCACGCCTCAAGGAGC* | | *TTCAGCGACACAAGATGCGA* |
| TFRC | NM_003234.4 | *GCAAGTAGATGGCGATAACAG* | | *GACGATCACAGCAATAGTCCC* |
| HMOX1 | NM_002133.3 | *CTTTCAGAAGGGCCAGGTGA* | | *GTAGACAGGGGCGAAGACTG* |
