# Supplementary material for: Iron overload inhibits BMP/SMAD and IL-6/STAT3 signaling to hepcidin in cultured hepatocytes
Source: PLoS One. 2021 Jun 23;16(6):e0253475. doi: 10.1371/journal.pone.0253475 (PMC8221488; doi:10.1371/journal.pone.0253475)
Supplement: S1 Raw images — (PDF) [file pone.0253475.s004.pdf]

All ladder labels are in kDa
All images captured by scanning
on a HP LaserJet Enterprise Flow MFP M630

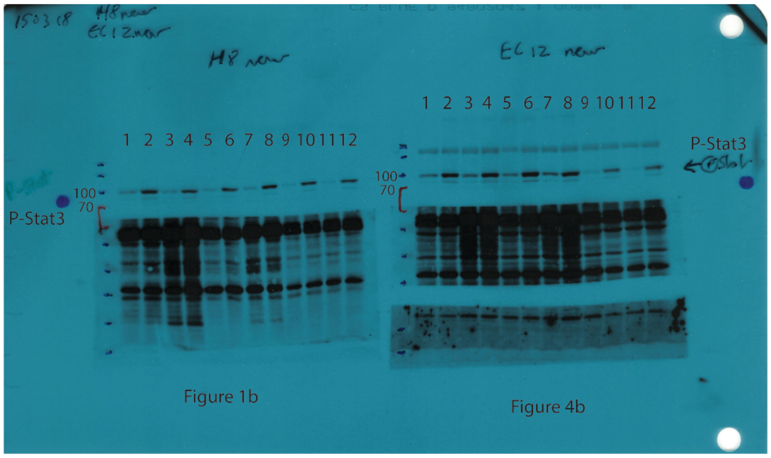

Legend: H8 + EC12
1 = Control
2 = IL6
3 = BMP6
4 = IL6 + BMP6
5 = DFO
6 = DFO + IL6
7 = DFO + BMP6
8 = DFO + IL6 + BMP6
9 = DFO + FAC
10 = DFO + FAC + IL6
11 = DFO + FAC + BMP6
12 = DFO + FAC + IL6 + BMP6

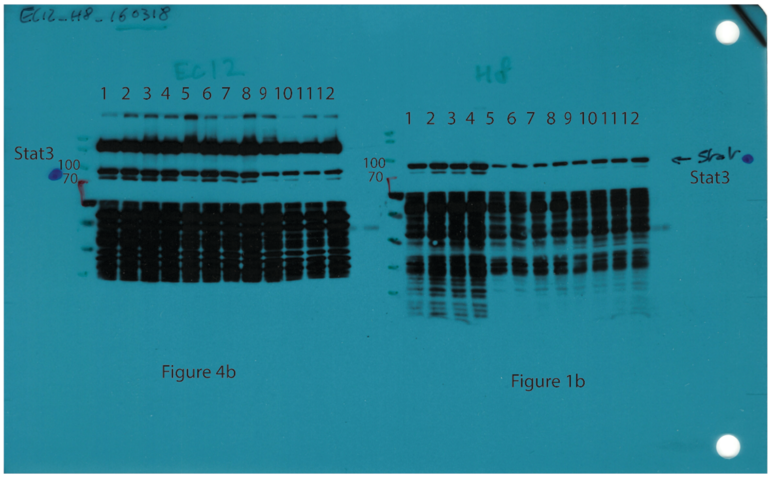

Legend: H8 + EC12
1 = Control
2 = IL6
3 = BMP6
4 = IL6 + BMP6
5 = DFO
6 = DFO + IL6
7 = DFO + BMP6
8 = DFO + IL6 + BMP6
9 = DFO + FAC
10 = DFO + FAC + IL6
11 = DFO + FAC + BMP6
12 = DFO + FAC + IL6 + BMP6

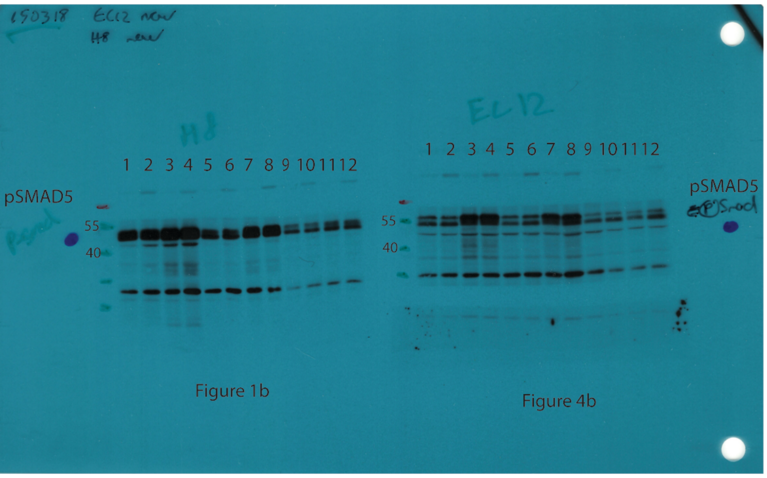

Legend: H8 + EC12
1 = Control
2 = IL6
3 = BMP6
4 = IL6 + BMP6
5 = DFO
6 = DFO + IL6
7 = DFO + BMP6
8 = DFO + IL6 + BMP6
9 = DFO + FAC
10 = DFO + FAC + IL6
11 = DFO + FAC + BMP6
12 = DFO + FAC + IL6 + BMP6

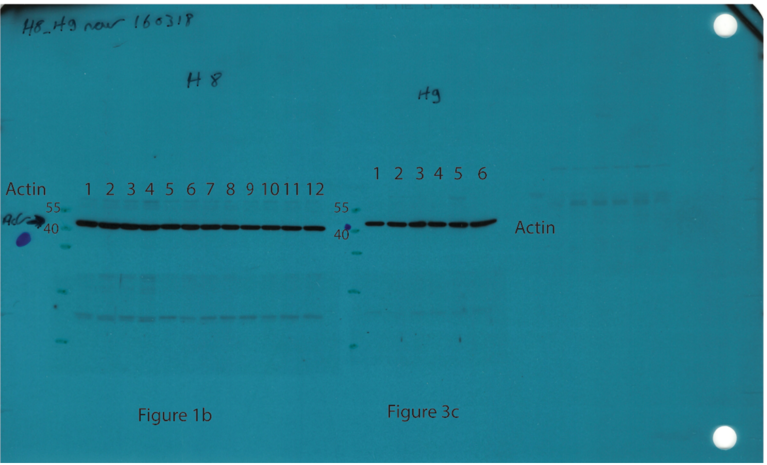

Legend: H8
1 = Control
2 = IL6
3 = BMP6
4 = IL6 + BMP6
5 = DFO
6 = DFO + IL6
7 = DFO + BMP6
8 = DFO + IL6 + BMP6
9 = DFO + FAC
10 = DFO + FAC + IL6
11 = DFO + FAC + BMP6
12 = DFO + FAC + IL6 + BMP6

Legend: H9
1 = Control
2 = BMP6
3 = BMP6 + FAC 2uM
4 = BMP6 + FAC 5uM
5 = BMP6 + FAC 20uM
6 = BMP6 + FAC 50uM

All ladder labels are in kDa

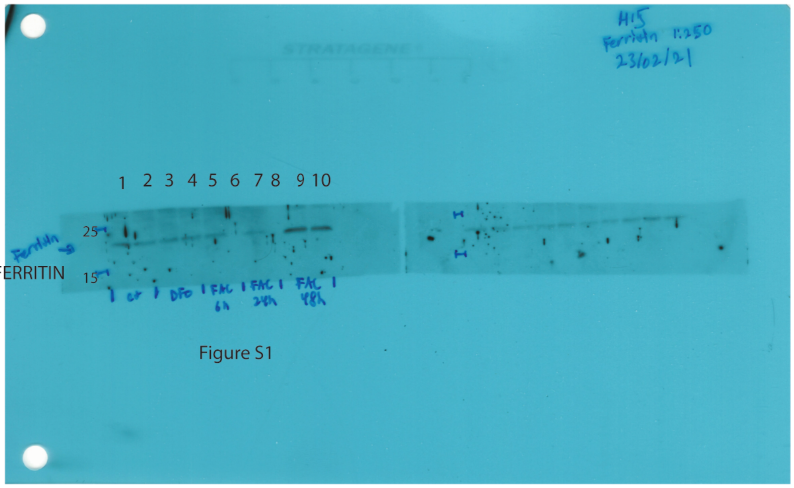

Figure S1

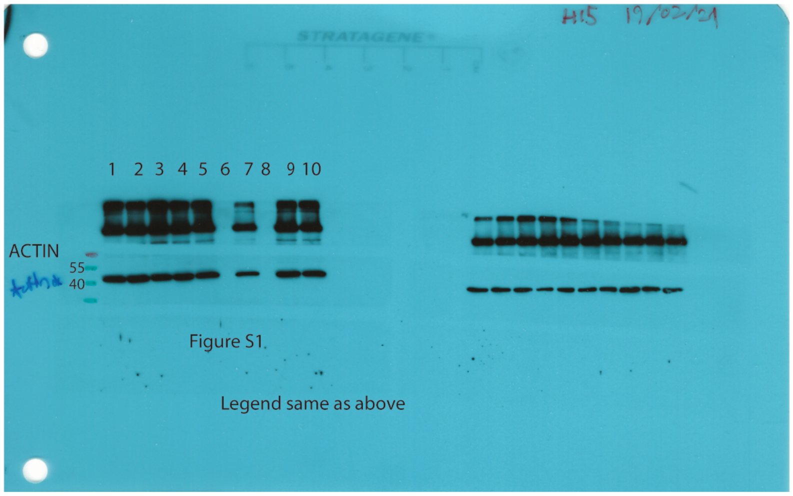

Figure S1

Legend same as above

Legend: EC12
1 = Control
2 = IL6
3 = BMP6
4 = IL6 + BMP6
5 = DFO
6 = DFO + IL6
7 = DFO + BMP6
8 = DFO + IL6 + BMP6
9 = DFO + FAC
10 = DFO + FAC + IL6
11 = DFO + FAC + BMP6
12 = DFO + FAC + IL6 + BMP6

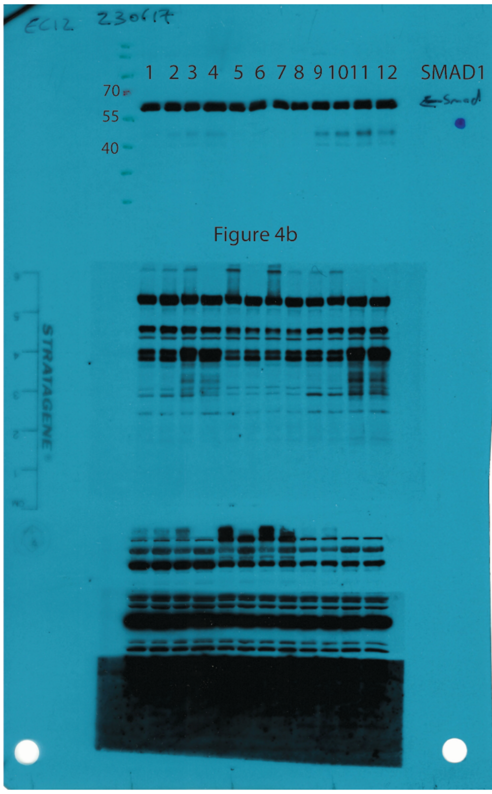

Figure 4b

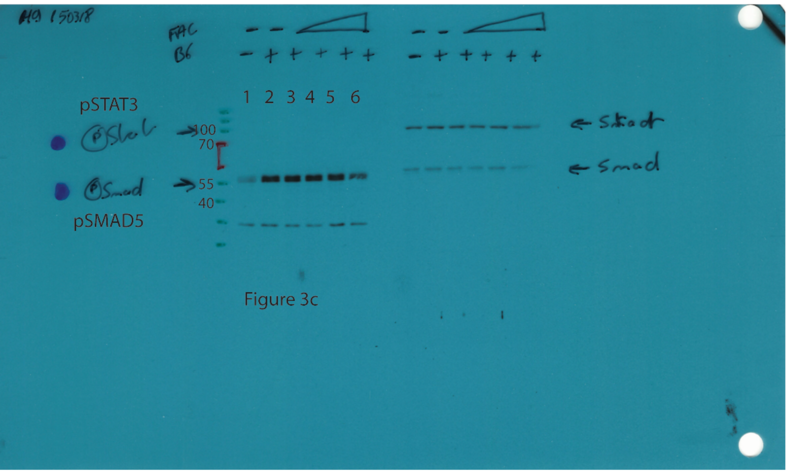

Figure 3c

Legend: H9
1 = Control
2 = BMP6
3 = BMP6 + FAC 2uM
4 = BMP6 + FAC 5uM
5 = BMP6 + FAC 20uM
6 = BMP6 + FAC 50uM

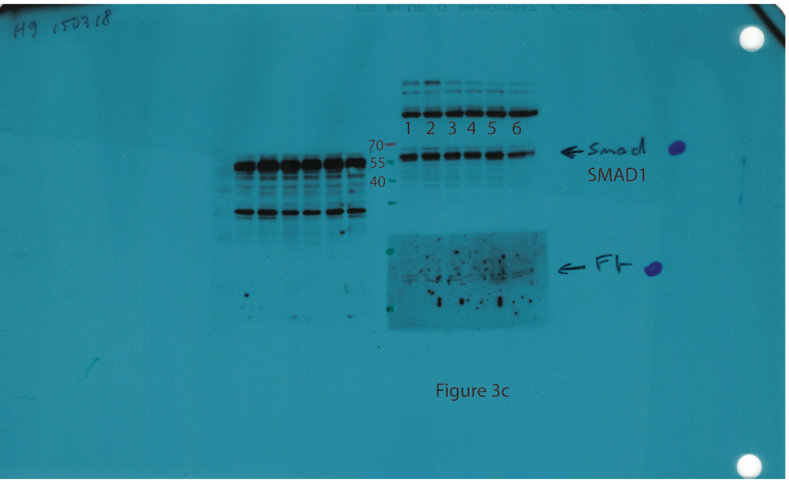

Figure 3c

Legend: H9
1 = Control
2 = BMP6
3 = BMP6 + FAC 2uM
4 = BMP6 + FAC 5uM
5 = BMP6 + FAC 20uM
6 = BMP6 + FAC 50uM

All ladder labels are in kDa

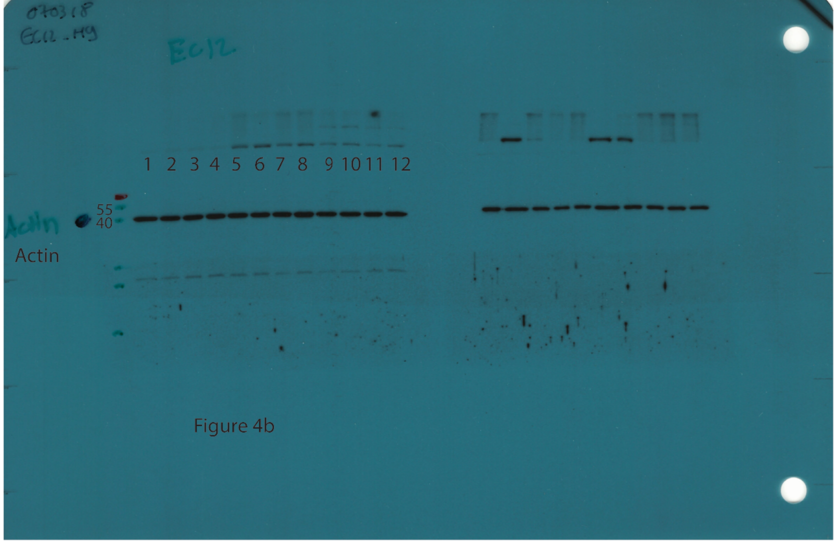

Legend: EC12
1 = Control
2 = IL6
3 = BMP6
4 = IL6 + BMP6
5 = DFO
6 = DFO + IL6
7 = DFO + BMP6
8 = DFO + IL6 + BMP6
9 = DFO + FAC
10 = DFO + FAC + IL6
11 = DFO + FAC + BMP6
12 = DFO + FAC + IL6 + BMP6

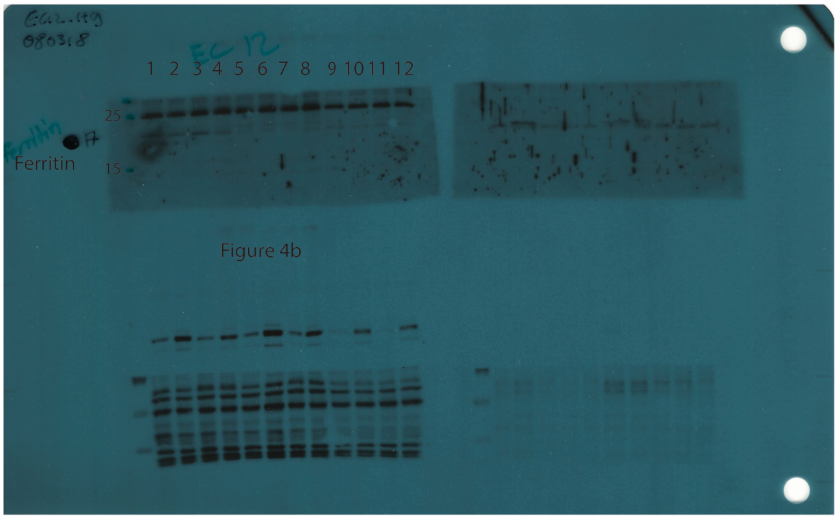

Legend: EC12
1 = Control
2 = IL6
3 = BMP6
4 = IL6 + BMP6
5 = DFO
6 = DFO + IL6
7 = DFO + BMP6
8 = DFO + IL6 + BMP6
9 = DFO + FAC
10 = DFO + FAC + IL6
11 = DFO + FAC + BMP6
12 = DFO + FAC + IL6 + BMP6

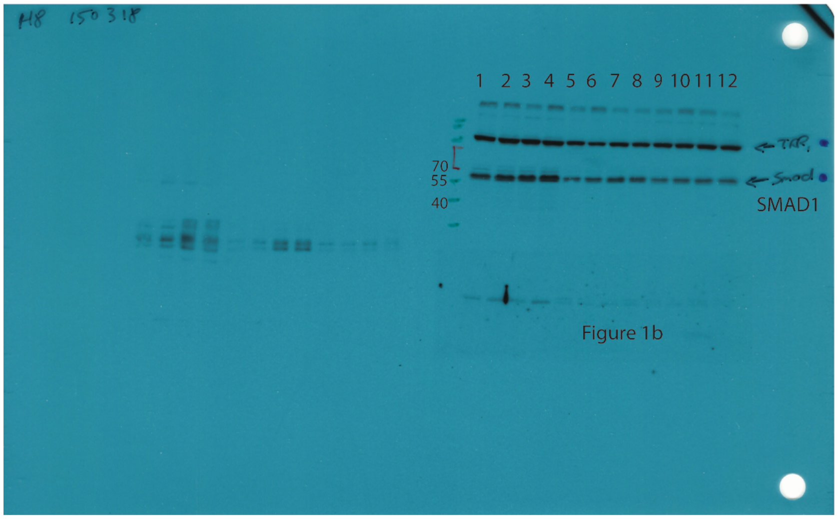

Legend: H8
1 = Control
2 = IL6
3 = BMP6
4 = IL6 + BMP6
5 = DFO
6 = DFO + IL6
7 = DFO + BMP6
8 = DFO + IL6 + BMP6
9 = DFO + FAC
10 = DFO + FAC + IL6
11 = DFO + FAC + BMP6
12 = DFO + FAC + IL6 + BMP6

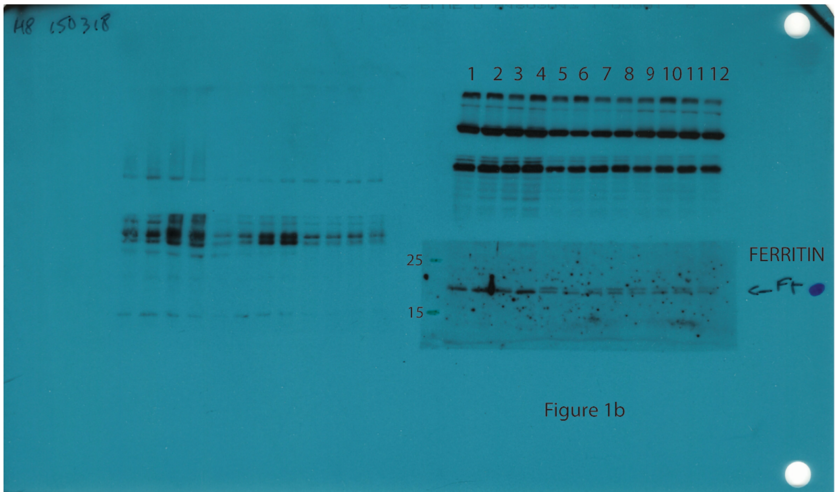

Legend: H8
1 = Control
2 = IL6
3 = BMP6
4 = IL6 + BMP6
5 = DFO
6 = DFO + IL6
7 = DFO + BMP6
8 = DFO + IL6 + BMP6
9 = DFO + FAC
10 = DFO + FAC + IL6
11 = DFO + FAC + BMP6
12 = DFO + FAC + IL6 + BMP6
